# Doctor and Patient

**Published:** 1921-07-23

**Authors:** 


### THE EDITOR'S LETTER-BOX.

**Correspondence on all subjects is invited, but we cannot in any way be responsible for the opinions expressed by our correspondents, who must give their name and address as a guarantee of good faith, but not necessarily for publication. Correspondents are reminded that brevity of style and conciseness of statement greatly facilitate early insertion.**

### Doctor and Patient.

*To the Editor of* The Hospital.

Sir,—Permit me to thank my critics, and to answer them. I do not know how far the writers represent lay or professional opinion, but, presumably, "F. Alex." is a doctor. In nicely chosen words he accuses me of culpable ignorance. And, strange as it may appear, I have regarded myself as rather on the alert, which fact, I venture to suggest, demonstrates that the propaganda which "F. Alex." believes to exist is ineffectual. Now what I have to charge your correspondent with is vagueness. He deals mainly in generalities. His reference to the daily press is not quite to the point. Such articles as may from time to time appear there represent lay effort, even if the writers are doctors, and they are spasmodic, unorganised, and usually anonymous. In one instance only is "F. Alex." specific, and it is in this connection that I desire to say a word. He asks if I read The Hospital.

My answer is that I have derived a great deal of useful knowledge from this journal. But—and here is the point—The Hospital articles are not written for such as myself. It is not a domestic journal. I have read vastly more appealing articles in American health journals written exclusively for the laity. Hence the appeal which I made that The Hospital should be so conducted as to find a place in every household. I have read articles from *The Lancet* obviously intended for the education of the laity, but I read them in the daily press as quotations. I do not call this effectual propaganda. Does "F. Alex." ?

May I offer "F. Alex." an illustration of a layman struggling in the dark? I am the layman, and what I am about to state may make me look ridiculous in the professional mind. I am, nevertheless, prepared to run the gauntlet. Within my limited circle of acquaintances I know of a large number of people (1) who suffer from rheumatism, and (2) who have had to undergo operations for appendicitis. I have made it my business to inquire, merely as an experimentalist, whether the sufferers were or were not water-drinkers. I found that an overwhelming majority of the people referred to are not water-drinkers. I mention this with all the modesty of admitted ignorance I make absolutely no pretence to scientific knowledge. I do not know whether the profession will sympathise or scoff. But if, for argument's sake, I happen to be right, I want friend "F. Alex." to realise that I have never yet read an article dealing thoroughly with the subject in any lay or professional publication. I venture to challenge "F. Alex." Is water-drinking a preventive of disease, or is it not? And if it is, can he point out to me any propaganda on the subject which in my ignorance I may have missed?

IERNE.

